# Genetic specialization of key bifidobacterial phylotypes in multiple mother–infant dyad cohorts from geographically isolated populations

**DOI:** 10.3389/fmicb.2024.1399743

**Published:** 2024-07-03

**Authors:** Sainaiwaer Aihetanmu, Zhixuan Liang, Xueling Zhang, Baolong Luo, Huimin Zhang, Jian Huang, Fengwei Tian, Hailong Sun, Yongqing Ni

**Affiliations:** ^1^School of Food Science and Technology, Shihezi University, Shihezi, China; ^2^School of Food Science and Technology, Jiangnan University, Wuxi, China

**Keywords:** *Bifidobacterium*, mother–infant dyad, multilocus sequence typing, ethnicity, probiotic

## Abstract

Little has been known about symbiotic relationships and host specificity for symbionts in the human gut microbiome so far. *Bifidobacteria* are a paragon of the symbiotic bacteria biota in the human gut. In this study, we characterized the population genetic structure of three bifidobacteria species from 58 healthy mother–infant pairs of three ethnic groups in China, geographically isolated, by Rep-PCR, multi-locus sequence analysis (MLSA), and *in vitro* carbohydrate utilization. One hundred strains tested were incorporated into 50 sequence types (STs), of which 29 STs, 17 STs, and 4 STs belong to *B. longum* subsp. *longum*, *B. breve*, and *B. animalis* subsp. *lactis*, respectively. The conspecific strains from the same mother–child pair were genetically very similar, supporting the vertical transmission of *Bifidobacterium* phylotypes from mother to offspring. In particular, results based on allele profiles and phylogeny showed that *B. longum* subsp. *longum* and *B. breve* exhibited considerable intraspecies genetic heterogeneity across three ethnic groups, and strains were clustered into ethnicity-specific lineages. Yet almost all strains of *B. animalis* subsp. *lactis* were incorporated into the same phylogenetic clade, regardless of ethnic origin. Our findings support the hypothesis of co-evolution between human gut symbionts and their respective populations, which is closely linked to the lifestyle of specific bacterial lineages. Hence, the natural and evolutionary history of *Bifidobacterium* species would be an additional consideration when selecting bifidobacterial strains for industrial and therapeutic applications.

## Introduction

The mammalian intestine is home to a complex and dynamic community of trillions of microorganisms, comprising hundreds in species (Derrien et al., 2019). Modern humans and ancestral animals have evolved since the most ancient times with a commensal microbiota (Moeller et al., 2016; Groussin et al., 2020). However, only gut bacteria that interact closely with the host immune system and produce a wide range of secondary metabolites beneficial for host health are defined as host symbionts (or mutualists) (Douglas and Werren, 2016). Gut symbionts rely on their hosts to maintain a stable habitat environment and access nutrients. The presence of such intimate interactions in the animal kingdom is used as evidence to advocate that animals and their gut symbionts have co-evolved (Oh et al., 2010; Groussin et al., 2020; Moeller and Sanders, 2020).

To date, such strict and typical co-evolution has been intensively described in some insect species that co-evolve with their endosymbionts (Gil et al., 2004; Koch et al., 2013; Eleftherianos et al., 2018). In the gut microbiome of great-apes, certain lineages of *Bacteroides* and *Bifidobacterium* were substantiated to co-evolve with their hosts (Moeller et al., 2016). *Limosilactobacillus reuteri*, as a typical host-adapted model lactobacillus, evolved a high degree of host specialization and clustered cohesively by host animal origin in the phylogenetic tree (Oh et al., 2010). However, this is not the case for all symbiont bacteria in the gut microbiome of mammalian animals. Symbiont bacteria have nomadic and vertebrate host-adapted lifestyles, which largely determine the closeness of their relationship with their animal hosts. To date, little has been known about symbiotic relationships and host specificity for specific taxa in the human gut microbiome.

*Bifidobacteria* are one of the first colonizers of the infant intestinal microflora, represented mainly by *B. breve*, *B. bifidum*, and *B. longum* subsp. *infantis* (Milani et al., 2017; Wong et al., 2020). *Bifidobacterium* lineages in the infant gut are vertically transferred from the maternal gut through breastfeeding, although the adult gut microbiome is featured by adult-type bifidobacteria species, including *B. adolescentis*, *B. longum* subsp. *longum*, *B. pseudocatenulatum,* and *B. catenulatum* (Turroni et al., 2018; Wong et al., 2020). Therefore, strains of both infant-type and/or adult-type bifidobacteria species from the same mother–infant pair would experience the same selective pressure and share similar genetic architecture and phenotypic characteristics.

In the present study, we isolated and distinguished *Bifidobacterium* strains from 58 healthy mother–infant pairs of three ethnic groups, including Xinjiang Uygur, Gansu Han, and Hainan Li of China, the habitats of which are separated by more than 2,000–4,000 km, with contrasting geographical and climatic environments. The purpose of our study was to illuminate whether key HRB phylotypes strictly follow vertical transmission from mother to infant. To achieve this goal, we focused on the distribution of the multilocus sequence types (STs) of strains of three bifidobacteria phylotypes (*B. longum* subsp. *longum*, *B. breve,* and *B. animalis* subsp. *latics*) most commonly present in three ethnic mother–infant pair cohorts. We tried to verify their ethnic specificity by comparing the genomic architecture and phenotypic traits of *Bifidobacterium* strains from multiple mother–infant cohorts that were geographically isolated.

## Materials and methods

### Subject recruitment and sample collection

**Figure 1:**
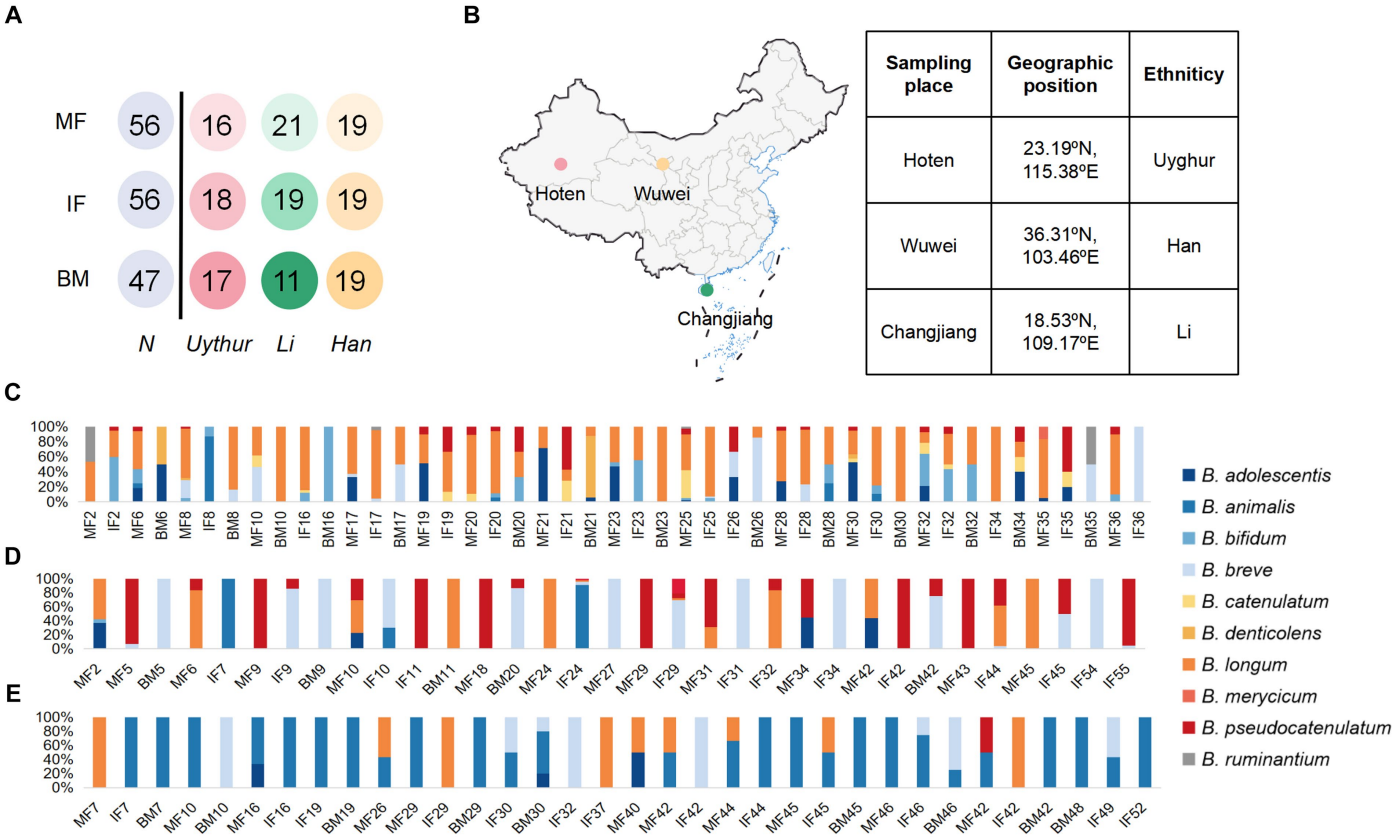
Schematic of the sampling data and *Bifidobacterial* profiling of 58 mother–infant sample sets. **(A)** Schematic depicts the total number of mother fecal, breast milk, and corresponding infant fecal samples from whom data were available. MF, maternal feces; IF, infant feces; BM, breast milk. **(B)** The samples were collected in Hoten, Xinjiang (23.19°N, 115.38°E), Changjiang, Hainan (18.53°N, 109.17°E), and Wuwei, Gansu (36.31°N, 103.46°E) Province, P.R. China, respectively. Bar plot of bifidobacterium isolated from each mother–infant pair of three ethnic groups. **(C)** Uygur group, **(D)** Li group, and **(E)** Han group. The number on the horizontal axis represents the family number.

A total of 58 mother–infant pairs were recruited in this study: 18 were from Hoten, Xinjiang (23.19°N, 115.38°E), 21 from Changjiang, Hainan (18.53°N, 109.17°E), and 19 from Wuwei, Gansu (36.31°N, 103.46°E) (Figure 1). All volunteers recruited in this study were local inhabitants and retained the custom of strict intra-ethnic marriages from time immemorial. None of these volunteers had gastrointestinal diseases or were exposed to antibiotics for at least 3 months before sampling.

Fresh maternal and infant feces samples were collected with a sterile plastic spatula and frozen immediately. The first drop of breast milk was discarded. Then, the colostrum, transitional, or mature breast milk samples were collected in sterile tubes using sterile gloves. All samples were kept at −20°C before being delivered to the laboratory. This study was approved by the Ethics Committee of the First Affiliated Hospital, Shihezi University School of Medicine, and informed consent was obtained from all volunteers before enrolling in the study.

### Isolation and identification of *Bifidobacterium* species

Serial dilutions of the supernatants derived from fecal (10^−3^, 10^−4^, and 10^−5^) and breast milk samples (10^−1^ and 10^−2^) were inoculated onto *Bifidobacterium* selective agar [BSM, MRS containing 0.05% (v/w) L-cysteine hydrochloride, and mupirocin (50 mg/L)] (Turroni et al., 2009) and MAN agar [Wilkins-Chalgren agar supplemented with soya peptone (5 g/L), L-cysteine (5 g/L), Tween 80 (1 mL/L), mupirocin (100 mg/L), and glacial acetic acid (1 mL/L)] (Vlkova et al., 2015) with two independent biological replicates at 37°C for 48–72 h. The DG500 anaerobic system was used for the anaerobic cultivation of plates filled with a gas mixture (80%N_2_, 10%CO_2_, and 10%H_2_). Eight colonies per plate, showing different morphology on the medium, were streaked for purity on MAN agar. Microscope was used to verify purity, and pure cultures were stored in MRS broth containing 25% glycerol at −80°C.

DNA from pure cultures was extracted through rapid mechanical cell lysis, as described in a previous study (Ventura et al., 2001). Identification of the strains at the species level was carried out by PCR sequencing of the *groEL* gene using primers Bif-*groEL*-F and Bif-*groEL*-R (Hu et al., 2017). Amplicons were verified by electrophoresis in 1.2% (w/v) agarose gels. Amplicons with bright bands at 500 bp were considered potential (suspected) bifidobacteria. Next, potential bifidobacterial isolates from all samples were screened and grouped using repetitive element polymerase chain reaction (rep-PCR) fingerprints for cost-effective speciation and typing. Two primers (GTG)_5_ (5′-GTGGTGGTGGTGGTG-3′) and BOXAIR (5’-CTACGGCAAGGCGACGCTG ACG-3′) were used for rep-PCR with their optimal PCR program (Jarocki et al., 2016). GelCompar II v6.0 (Applied Math, Sint-Martens- Latem, Belgium) embedding Pearson’s similarity coefficient and unweighted pair group method with arithmetic mean (UPGMA) were adopted to group rep-PCR banding patterns. Finally, one to three representative strains were sequenced by GENEWIZ company (Jiangsu, China). Sequences were subjected to BLAST search to align sequences with the NCBI BLASTn database, and the identities of isolates were qualified based on the highest scores.

### Multilocus sequence typing (MLST) analysis

MLST was performed with seven housekeeping genes as previously described (Supplementary Table S1) (Ventura et al., 2006; Alexis et al., 2010; Makino et al., 2013; Zhang et al., 2015). PCR were carried out in 25 μL volumes containing 12.5 μL of Premix Taq, 0.5 μL of each primer (20 μM), 8.5 μL of double distilled water (ddH_2_O), and 3 μL of the respective template DNA (from the supernatants after DNA extraction, which equals approximately 30–60 ng of DNA). Each PCR cycling profile consisted of a cycle of 95°C for 5 min, followed by 30 cycles of 95°C for 30 s, 55°C for 30 s, 72°C for 1 min, and finally 1 cycle of 72°C for 8 min. PCR products were verified by electrophoresis in 1.2% (w/v) agarose gels and sequenced by GENEWIZ company (Jiangsu, China).

Sequence data of strains were edited and aligned using Chromas v2.6.6. Seven housekeeping gene sequences were downloaded as an MLST database established in BioNumerics v8.0 (Applied-Maths, Sint-Martens-Latem, Belgium). The allele numbers and STs were assigned using default parameters in BioNumerics v8.0. The minimum spanning tree (MST) analysis was constructed with Prim’s algorithm embedded in the BioNumerics software according to isolation sources and regions. Global Optimal eBURST analysis (goeBURST) was used to group and cluster STs to clonal complexes (CCs) based on their allelic profiles. The strains that shared a minimum of five out of the seven alleles were grouped in a CC. Split-treev4.0 software was used to evaluate the impact of recombination events on phylogeny, and split decomposition analysis was constructed. A Phi-test attached to this software was used to detect whether there was significant recombination in alleles. The linkage equilibrium between alleles was calculated with LIAN 3.0, and the standardized index of association (I^S^_A_) was detected.

### Host- and diet-derived carbohydrate utilization

One host-derived glycan (mucin) and eight diet-originated glycans were selected for carbohydrate utilization tests. Except for mucin, other commercial oligosaccharides were purchased from Yuanye Biotechnology Co., Ltd., Shanghai, China, including stachyose, raffinose, xylooligosaccharides (XOS), isomaltooligosaccharide (IMO), inulin, fructo-oligosaccharide (FOS), resistant starch (RS), and galactooligosaccharides (GOS). The mucin was extracted from porcine gastric. *Bifidobacterial* strains were cultivated anaerobically at 37°C for 18 h on MRS broth for two generations. Cultures were centrifuged at 8000 rpm for 5 min, washed and resuspended in PBS serving as seed cultures. The carbohydrate utilization of *Bifidobacterium* was investigated in semisynthetic MRS media supplemented with 1.0% (w/v) of a particular glycan. The media were inoculated with 1.5% of the seed cultures. The same concentration of glucose was used as the positive control, while the medium without sugar supplementation was used as the negative control. Bacterial growth was monitored at OD_600_ using a spectrophotometer at 48 h. Cultures were grown in biologically independent duplicates.

## Results

### Study cohorts

In this study, we enrolled 58 healthy mother–infant pairs from 3 ethnic groups (that is, the Uygur, Li, and Han) from 3 provinces in China. Of these, 18 mother–infant pairs from the Uygur ethnic group living in Hoten, Xinjiang, China, and 21 mother–infant pairs from the Li ethnic group living in Changjiang, Hainan, China, were compared with the 19 mother–infant pairs from the Han group living in Wuwei, Gansu, China. All volunteers recruited lived far away from the metropolitan areas with typical pastoral or farming lifestyles (Table 1). The diet of Uighur is low in fiber and rich in fat, sugar, and animal protein. The most common diet is beef, mutton, and milk. The Li diet is characterized by starch and plant polysaccharides and mainly includes fruits, seafood, and porridge. The Han mother–infant pair diet is rich in starch and fiber, consisting mainly of cereals and vegetables. All foods are sourced locally and processed manually. Fecal and breast milk samples were obtained. After excluding mother–infant pairs with missing data, 112 fecal and 47 breast milk samples were included for analysis (Figure 1).

**Table 1:** Characteristic data of the mother–infant pairs in this study.

Characteristics data	Values or (%)		
	Uyghur (*n* = 18)	Han (*n* = 19)	Li (*n* = 21)
Infant sex			
Male	8 (44.44%)	10 (52.64%)	9 (42.86%)
Female	10 (55.56%)	9 (47.36%)	12 (57.14%)
Infant age	6–12 months	6–14 months	1–4 months
Mother age (mean ± SD, range)	27.74 ± 5.10 (22–44)	28.74 ± 3.74 (23–35)	27.92 ± 4.05 (22–35)
Mother BMI (mean ± SD, range)	25.86 ± 4.25 (17.72–34.62)	21.61 ± 3.06 (16.61–25.63)	26.37 ± 5.91 (20.03–34.45)
Lifestyle	Pastoral	Farming	Farming
Breastfeeding time	6 months	< 4 months	>6 months
Antibiotics	Never used	Never used	Never used
Diet	low in fiber and rich in fat, sugar, and animal protein	low in fat, rich in starch and fiber	low in fat and rich in starch, plant polysaccharides

### Isolation and identification of bifidobacteria in the fecal/milk microbiota of mother–infant pairs from three ethnic groups

We assessed the composition of fecal/milk *Bifidobacterial* population of 58 corresponding mother–infant sets from three ethnic groups. A total of 643 *bifidobacterium* strains were divided into 10 species for the Uygur group, 356 into 7 species for Li, and 192 into 6 species for the Han group. For the Uygur group, bifidobacterial isolates could be isolated from 14 (93%), 15 (83.3%), and 14 (82.3%) samples of maternal feces, infant feces, and breast milk, respectively. *B. longum* were found at relatively high frequencies in all three ecosystems; however, fecal isolates comprised mainly *B. adolescentis and B. bifidum*, while *B. denticolens* predominated over *Bifidobacterium* spp. among breast milk isolates (Figure 1C). There are 356 bifidobacterial isolates were obtained from the Li ethnic group, including 13 (65%), 14 (73.7%), and 5 (45.5%) samples of maternal feces, infant feces, and breast milk, respectively. *B. pseudocatenulatum, B. breve,* and *B. animalis* were isolated mainly from fecal samples. Members of *B. breve* represented 83.9% of breast milk isolates (Figure 1D). The isolation frequencies of Han were lower those of the Uygur and Li groups, including 9 (47.36%), 14 (73.68%), and 9 (47.36%) samples of maternal feces, infant feces, and breast milk, respectively. Isolates of *B. animalis* were commonly found in both faces and breast milk. *B. breve* occurred frequently in infant feces, while *B. longum* were detected at relatively high frequencies in maternal feces (Figure 1E).

**Figure 2:**
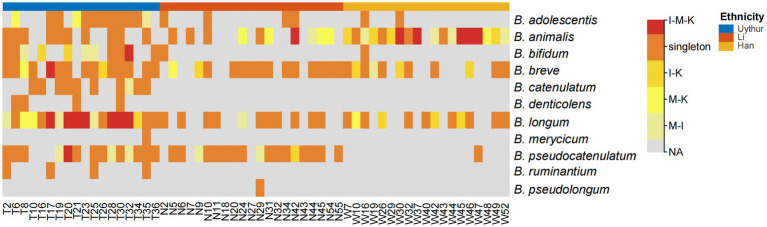
Heat map of bifidobacterial species that shared within mother–infant pairs. I, infant feces; M, mother feces; K, breast milk. I–M–K, bifidobacteria species appeared to be present in all infant feces, mother feces, and breast milk samples, and so on. Singleton, bifidobacteria species from only the mother or infant in each dyad. NA, no bifidobacteria species were obtained. The number on the horizontal axis represents the family number.

Albeit the difference in isolation frequency and species composition between maternal feces, infant feces, and breast milk, the species *B. longum*, *B. breve*, *B. animalis, B. adolescentis,* and *B. pseudocatenulatum* were commonly detected in multiple mother–infant dyads across three ethnic groups. Among them, *B. longum* had the highest co-occurrence rate in mother–infant dyads (41/58), followed by *B. breve* (40/58), *B. animalis* (37/58), and *B. pseudocatenulatum* (32/58) (Figure 2). *Bifidobacterium,* as obligate anaerobe, are unlikely to result from skin or environmental contamination. The existence of the same isolates within mother–infant dyads has confirmed the vertical transmission from mother to infant through breastfeeding. However, the hypothesis of mother–infant vertical transmission must be verified at the strain level. Therefore, a higher resolution method, MLST, was adopted to illuminate whether all HRB phylotypes strictly follow vertical transmission from mother to infant at the strain level.

### Comparison of MLST profiles of *Bifidobacterial* strains isolated from members of mother–infant pair from three ethnic groups

We performed our study on key bifidobacterial phylotypes which were frequently isolated from three ethnic groups or contained strains known for their probiotic effects, such as *B. longum*, *B. breve,* and *B. animalis*. Rep-BOXAIR-PCR and rep-(GTG)_5_-PCR were additionally conducted on all three species for cost-effective speciation and typing. A total of 435 isolates of *B. longum*, 167 isolates of *B. breve,* and 219 isolates of *B. animalis* from 58 mother–infant pairs were analyzed by rep-PCR. According to the rep-PCR band pattern, 100 representative isolates (*B. longum*, 37 isolates; *B. breve*, 33 isolates; and *B. animalis*, 30 isolates) were subsequently used for housekeeping gene sequencing (Supplementary Figure S1). Following sequencing and concatenated sequence analysis, 37 *B. longum* strains were identified as *B. longum* subsp. *longum* and 30 *B. animalis* strains were identified as *B. animalis* subsp*. lactis.*

**Figure 3:**
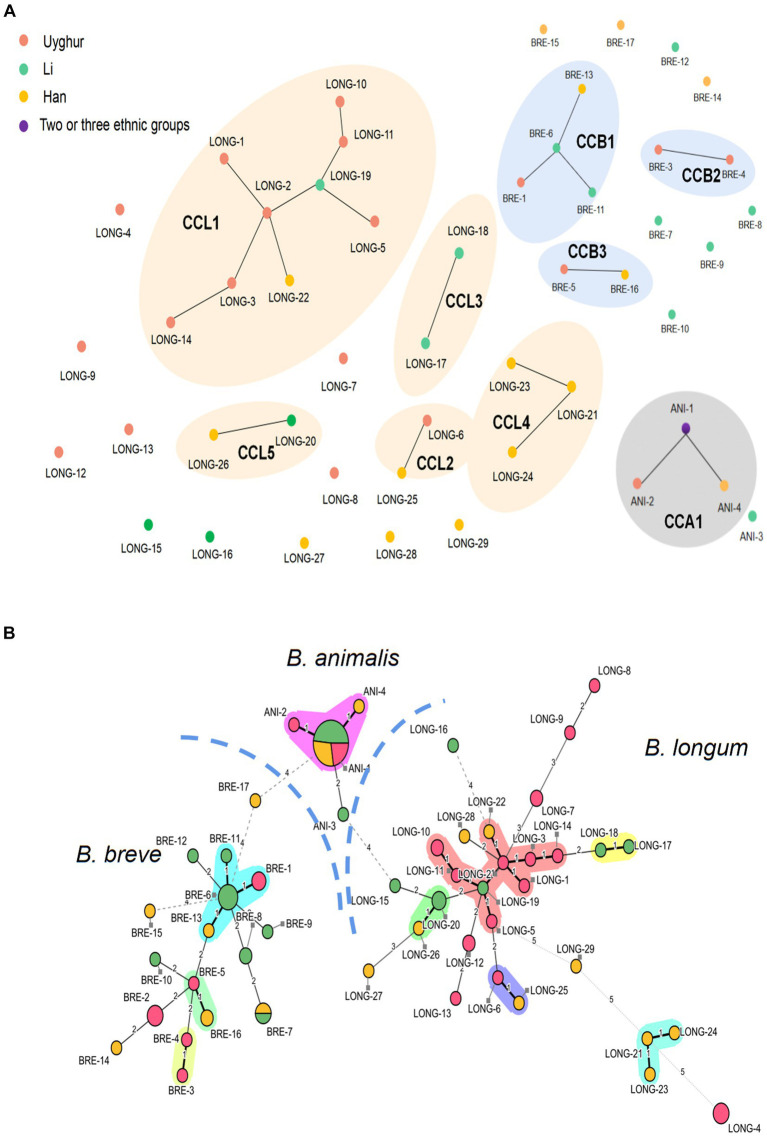
Comparison of MLST profiles of 100 bifidobacteria strains. **(A)** A diagram represents clonal relationships between STs was generated from goeBURST. **(B)** Minimum spanning tree of 100 *Bifidobacterium* strains based on MLST profiles according to the isolation source. Each filled circle corresponds to an ST. The color of the circle represents the source of isolation. Pink, Uygur; Green, Li; Yellow, Han; Purple, strains from two or more ethnic groups. Clonal complexes are represented by colored shaded areas.

Among the 100 *Bifidobacterium* strains, 50 distinct STs were identified as *B. longum* subsp. *longum*, 29 STs; *B. breve*, 17 STs; and *B. animalis* subsp*. lactis*, 4 STs, suggesting a high genetic diversity in key bifidobacterial phylotypes. A goeBURST analysis and MST were conducted to determine the relatedness between STs (Figures 3A,B). For *B. longum* subsp. *longum*, a total of 29 STs were divided into 5 CCs (CC_L1_–CC_L5_) and 11 singletons. Among the 14 STs from the Uygur group, 8 STs were grouped in 2 CCs and 6 were identified as single STs; among the 6 STs from the Li group, 4 STs were clustered in three CCs and 2 were identified as single; among the 9 STs from Han group, 6 STs were clustered in four CCs and 3 were identified as single. Three CCs (CC_B1_–CC_B3_) were detected in *B. breve* species, among which ST BRE-3 and BRE-4 from the Uygur group were grouped in CC_B2_ and BRE-1, BRE-6, BRE-11, and BRE-13 were clustered in CC_B1_. No coexistence of *B. longum* subsp. *longum* and *B. breve* strains with the same ST across ethnic groups were found except ST BRE-7. Almost all *B. animalis* subsp*. lactis* strains (28/30) were incorporated in the same CC, no matter what ethnicity.

**Figure 4:**
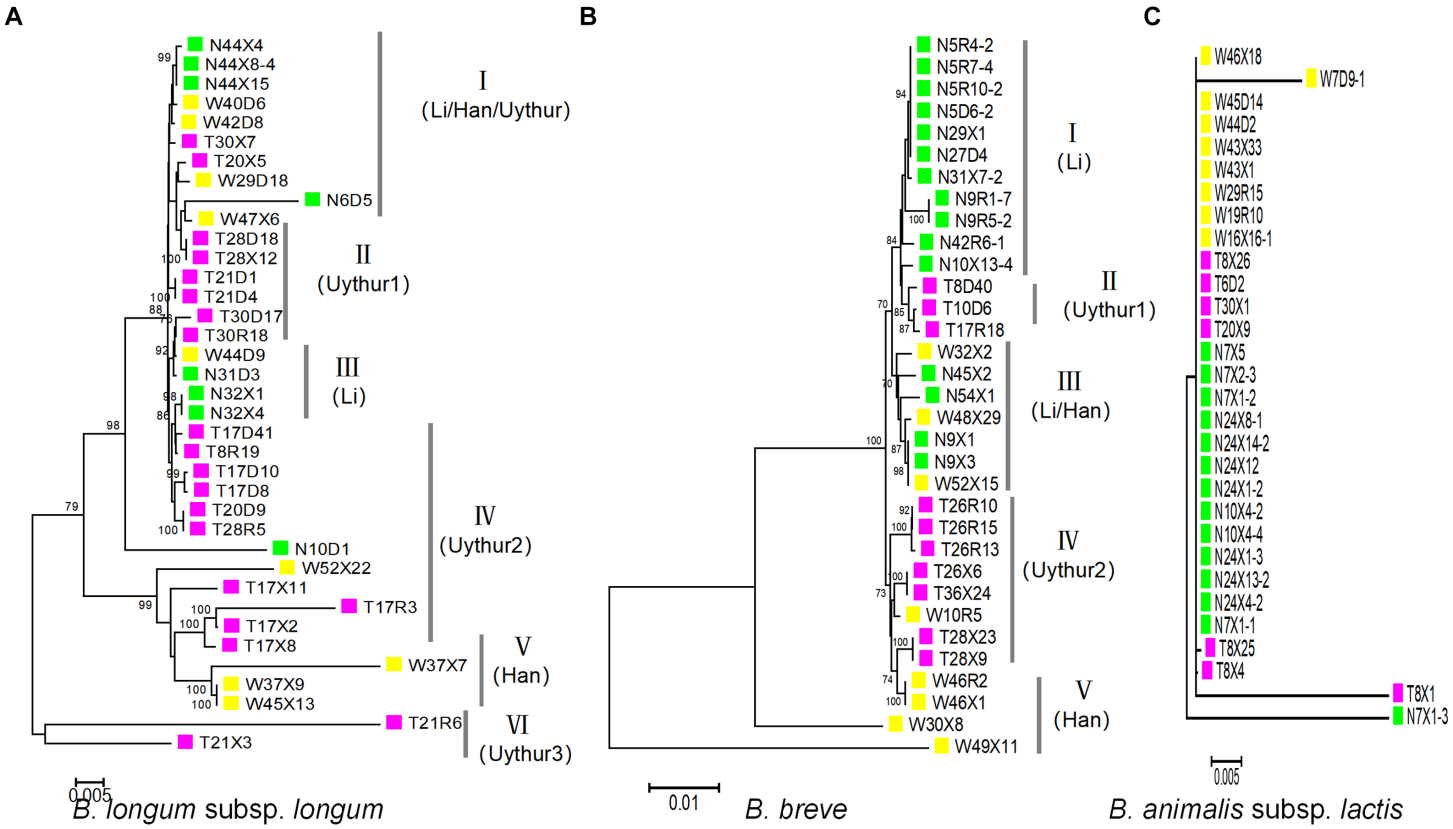
Maximum-likelihood phylogenetic tree obtained from the concatenated nucleotide sequence of 100 *Bifidobacterium* strains. For the ML phylogenetic trees, MEGA X software was used with the GTRGAMMAIX model. The number of bootstrap replicates was 1,000 and visualized using ITOL. **(A)** *B. longum* subsp. *longum*, **(B)** *B. breve*, and **(C)** *B. animalis* subsp*. lactis.*

A maximum-likelihood (ML) trees were built from the concatenated sequences to infer evolutionary relationships within *B. longum* subsp. *longum*, *B. breve*, and *B. animalis* subsp. *Lactis* (Figure 4). The analysis revealed six subpopulations in *B. longum* subsp. *longum* strains and five in *B. breve* that was host (ethnic) specific. For *B. longum* subsp. *longum*, subpopulations II, IV, and VI contain strains from the Uyghur ethnic group, subpopulation III contains strains from the Li ethnic group, and subpopulation V contains strains from the Han ethnic group. For *B. breve*, subpopulation I contains strains from the Li ethnic group, subpopulations II and IV contain strains from the Uyghur ethnic group, and subpopulation V contains strains from the Han ethnic group. The results indicate that the strains from same ethnicity are more likely to tightly assemble together are incorporated into same phylogenetic clade, while strains from diverse ethnic group showed lower relatedness. Although the subpopulation has reflected some ethnic specificity, not all strains of the subpopulations were incorporated into discrete phylogenetic clade. For example, subpopulation III of *B. breve* comprised strains from both the Li and Han groups. In general, both the MST and the concatenated sequence analysis support the hypothesis that ethnic specificity to a large degree (Figures 4A,B).

For *B. animalis* subsp. *lactis,* we surprisingly found that almost all *B. animalis* subsp. *lactis* strains had the same ST and were grouped in same subpopulation (CC), regardless of ethnicity (28/30) (Figure 4C).

### Influence of recombination on the population structure of key bifidobacterial phylotypes

**Figure 5:**
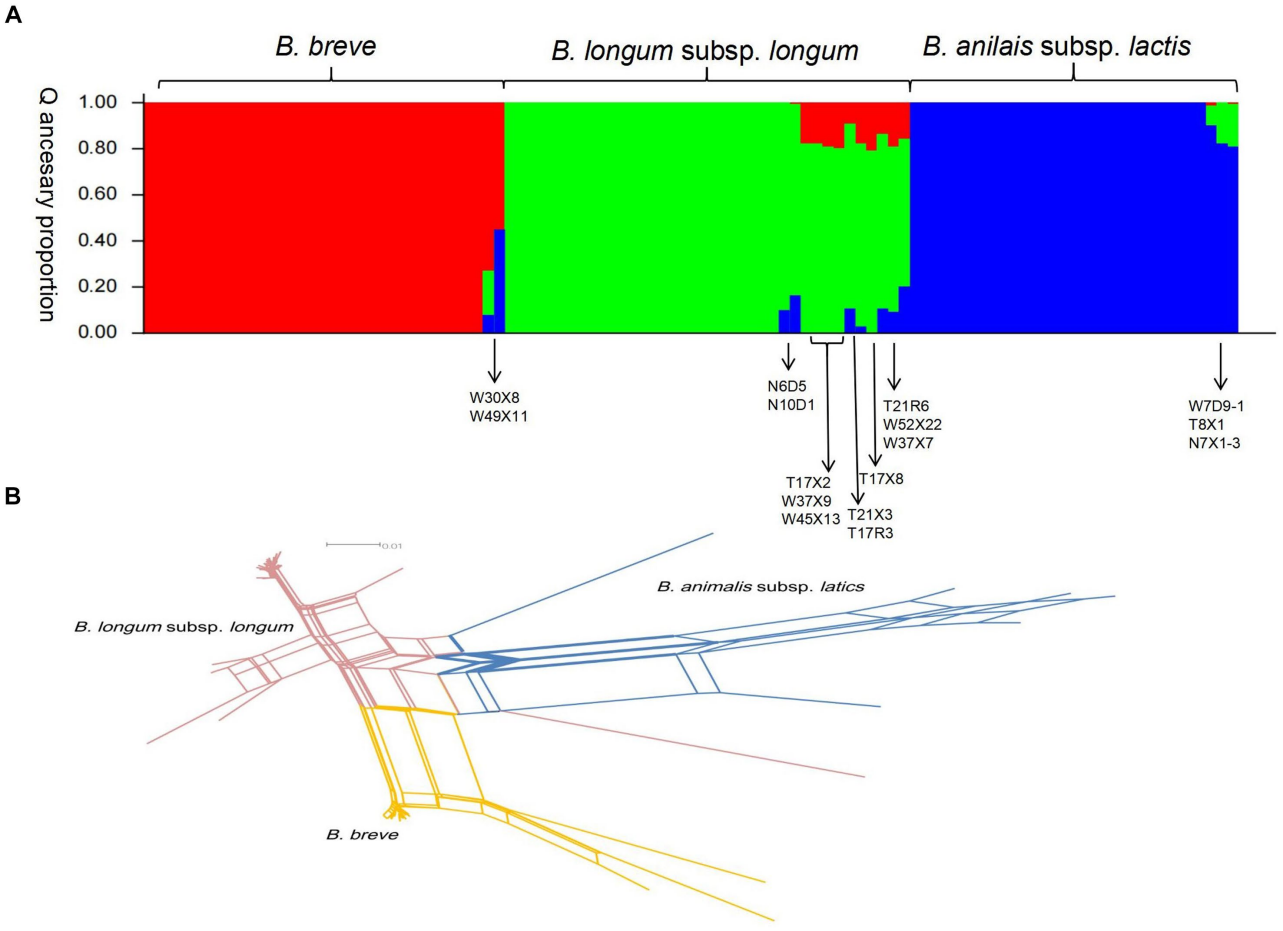
Recombination analysis of 100 strains of bifidobacteria. **(A)** Structure analysis. The plot shows one vertical line for each strain, and the length of the colored segments indicates the proportions of nucleotides from each of the three ancestral populations. **(B)** Combined split-decomposition analysis based on concatenated sequences of all 7 MLST loci.

Compared to point mutations, recombination has contributed more to strain diversification. To assess the interspecies recombination in key bifidobacterial phylotypes, we constructed the NJ tree of individual housekeeping genes (Supplementary Figure S2). The phylogenetic positioning of most strains in all individual genes was the same. However, we also found incongruent topologies in the NJ tree. These incongruent phylogenetic topology observations may be caused by interspecies homologous recombination. Structure v2.3.4 is also used to detect more subtle recombination events. Multiple runs with K values from 2 to 7 showed maximal posterior probability at K = 3. Three major ancestral populations were recognized (corresponding to three colors in Figure 5A), and little admixture of ancestral sources was found between the three key bifidobacterial phylotypes. The admixture may be a factor that contributed to the low bootstrap value in ML topology. For example, the nucleotide distance was long between T21R6, T21X3, and other isolates on the *B. longum* subsp. *longum* ML topology. Moreover, we found that strains within phylotypes (species) were more homogenous.

We next investigate the recombination events within key bifidobacterial phylotypes. First, we used LIAN-linkage v3.0 software to evaluate the level of linkage disequilibrium between 7 alleles of 100 representative isolates. In clonal populations, where mutations predominate in generating genetic variation, the I_A_^S^ values (the standardized index of association) of seven gene segments were significantly different from zero (linkage disequilibrium). In non-clonal populations, where recombination predominates, the I_A_^S^ values (the standardized index of association) of seven gene segments are close to zero (linkage equilibrium). For *B. longum* subsp. *longum* isolates, the I_A_^S^ value of seven gene loci in this study was 0.3023 (*p* < 0.01), demonstrating a tendency of linkage disequilibrium among the seven loci. Clonal structures were present among this set of *B. longum* subsp. *longum* isolates. For *B. breve* and *B. animalis* subsp*. lactis*, the I_A_^S^ values of 7 housekeeping genes were 0.0979 (*p* < 0.01) and 0.0695 (*p* < 0.01), respectively, indicating that there was obvious free recombination between alleles (linkage equilibrium). However, the I_A_^S^ value of the 100 representative isolates was 0.6349 (*p* < 0.01), demonstrating a tendency of linkage disequilibrium between seven gene segments (significantly different from zero, linkage disequilibrium). Second, a split decomposition analysis was performed. In this study, obvious parallelogram structures were found in all alleles, indicating that these genes had been affected by intergenic recombination in the evolution process (Supplementary Figure S3 and Supplementary Table S2). Parallelogram-shaped structures were also found in split graphs of concatenated sequences of the seven loci. The *p* value detected by the phi-test for the 7 individual MLST loci and concatenated sequences all<0.001, indicating recombination events occurred within and across 7 loci (Figure 5B).

On the whole, recombination events were frequently found within species but limited across species and genetic recombination occurred in all representative isolates tested during evolution.

### Analysis of carbohydrate utilization by *Bifidobacteria*

**Figure 6:**
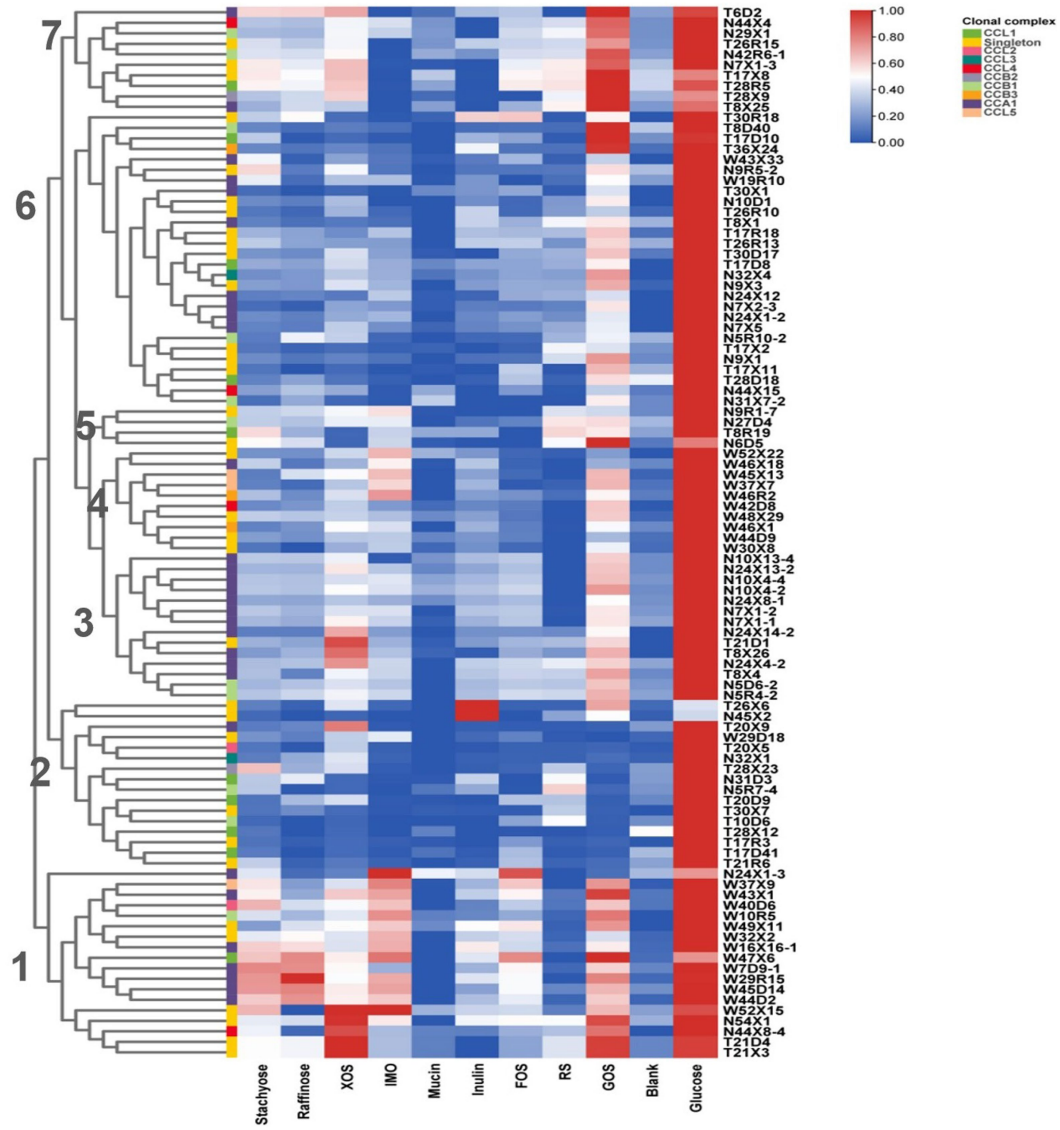
Growth of *Bifidobacteria* isolates on polysaccharides. Isolates were cultured anaerobically at 37°C in an MRS medium supplemented with individual polysaccharides as the sole carbon source. OD_600_ was measured at 0 and 48 h. Data indicate mean ± SD, and each condition was tested in duplicate. The heatmap shows the mean of the highest OD for each condition of each experiment. *Bifidobacteria* clonal complexes are indicated above the heatmap.

Continuously metabolizing a variety of carbohydrates derived from diet enables Human-Residential *Bifidobacteria* (HRB) to adapt and colonize in human host-associated niches. In our study, *in vitro* growth assays were conducted for 100 representative isolates, in which 9 common polysaccharides, including host- and plant-derived glycans, were utilized as the sole carbon source. As shown in Figure 6, most of the tested *Bifidobacterial* strains displayed vigorous growth in some plant-derived glycans, such as GOS. Surprisingly, none of the isolates grew well on host-derived glycans mucin (OD_600_≦0.349 ± 0.004). Notably, according to the glycan metabolic profile, we found similar fermentation capabilities of strains from the same ethnic group. Seven metabolic clusters were found among the 100 *Bifidobacterium* strains across three ethnic groups. Subclusters 1 and 4 contained the isolates almost from the Han group, clusters 2 contained the isolates almost from the Uyghur group, and clusters 3 and 6 contained the isolates both from Uyghur and Li groups. With respect to the source of the strains, *Bifidobacterial* strains from the same mother–infant pairs or same individual showed very similar metabolic profiles, reflecting in inter-strain very close phylogenetic relationships, such as T21D4 and T21X3; N5D6–4 and N5R4–2; T17D41 and T17R3; N10X4–2 and N10X4–4. Moreover, compared to *B. longum* subsp. *longum* and *B. breve*, more conservative carbohydrate utilization profiles were found in members of *B. animalis* subsp. *lactis.*

## Discussion

Accumulating evidence highlights that breast milk is an important intermediary of *Bifidobacterium* for the newborn gut, and *Bifidobacterium* strains in the maternal gut are vertically transferred to the infant’s gut through breastfeeding (Fehr et al., 2020; Lawson et al., 2020). However, these studies were conducted on a limited number of subjects (mother–infant pairs). In this study, we isolated *Bifidobacteria* strains from 58 mother–infant pairs across three ethnic groups and selected key bifidobacterial phylotypes for analyzing population structure and phylogeny to explore their host-specificity.

In our study, *B. longum* subsp. *longum*, *B. adolescentis*, *B. pseudocatenulatum*, and *B. animalis* subsp*. lactis* were the most common species recovered from mother fecal samples, while *B. longum* subsp. *longum, B. animalis* subsp*. lactis,* and *B. breve* were the most prevalent phylotypes in infant fecal samples. The most frequently isolated species in breast milk samples were *B. breve*, *B. animalis* subsp*. lactis*, *B. longum* subsp. *Longum,* and *B. denticolens*. As shown in Figure 2, *B. longum* subsp. *longum*, *B. breve*, *B. animalis* subsp*. lactis*, *B. adolescentis,* and *B. pseudocatenulatum* were detected in all three ecosystems of 58 matched mother–infant dyads, of which *B. longum* subsp. *longum*, *B. breve,* and *B. animalis* subsp*. lactis* with 70.69, 68.97, and 55.17% coincidence rates, respectively, exhibited the highest recovery frequency. To date, most studies based on culture techniques have reported very inconsistent results regarding the number and combination of *Bifidobacterium* phylotypes in breast milk and the infant gut across cohorts (Solis et al., 2010; Jost et al., 2013, 2014). According to our previous study on the occurrence of *Bifidobacterium* phylotypes in mother–milk–infant triads by *groEL* gene sequencing, 13 well-known *Bifidobacterium* species or subspecies were determined in a mother–infant cohort, of which 7 well-known *Bifidobacterium* species or subspecies showed triadic synchronism (Yan et al., 2021).

Most recently, multiple studies have confirmed the co-occurrence of multiple bifidobacterium species among the maternal gut, breast milk, and infant gut (Bogaert et al., 2023; Garmaeva et al., 2024). The conspecific strains from the same mother–child pair were assigned to the same monophyletic group, and they are genetically and physiologically very similar. However, it is not clear whether the conspecific strains within mother–infant populations with similar lifestyles and the same geographies have more similar genetic and phenotypic characteristics than strains from other population. Therefore, the most prevalent species, *B. longum* subsp. *longum*, *B. breve,* and *B. animalis* subsp*. lactis,* were selected for the analysis of population genetic structure and phylogeny. Results based on allele profiles showed a certain degree of correspondence between *B. longum* subsp. *longum* and *B. breve* strain grouping and their sources (mother–infant pair cohorts). Strains of both *Bifidobacterium* species inhabiting the gut microbiome of different human populations (human residential) did form separately a stable sub-population or sub-lineage. So, our results support the hypothesis of co-evolution between gut symbionts and their respective populations. This phenomenon can be explained by a claim: that the genetics of host populations and different daily diets exert selective pressures on specific bacterial species in the gut microbiota, leading to intraspecific genetic divergence (Martino et al., 2018; Dapa et al., 2022). Indeed, in our study, the three ethnic groups rarely intermarry for approximately a thousand years, and their dietary customs and local foodstuffs vary greatly.

However, this is not the case for all symbiont bacteria in the gut microbiome of mammalian animals. Symbionts with different life histories exhibit different responses to the selective pressure of the host environment. Existing studies showed the disparity of ecological fitness and evolutionary patterns across different gut symbionts shared by humans and animals (Duar et al., 2017; Wong et al., 2020). In the gut microbiome of great-apes and human beings, certain lineages of *Bacteroides* and *Bifidobacterium* were substantiated to co-evolve with their hosts, reflecting strain-level co-adaptation between bacteria and their hosts (Moeller et al., 2016). In *Bacteroides fragilis*, a prevalent commensal in the large intestine of healthy people, an adaptive mutation was found to emerge frequently in Western, but not Chinese, microbiomes (Zhao et al., 2019). *Limosilactobacillus reuteri*, as a typical host-associated lactobacilli, has specialized in ecological niches that are associated with hosts and formed host-specific sublineages (Oh et al., 2010). By contrast, *Lactiplantibacillus plantarum* was proposed to be a nomadic lifestyle (Duar et al., 2017) and displays no obvious clustering by origin (Martino et al., 2018; Yu et al., 2021). In this study, we found that almost all strains of *B. animalis* subsp. *lactis* belong to the same ST or genotype regardless of their ethnic source, which coincided with the observation of Milani et al., who reported that the *B. animalis* subsp. *lactis* strain has a strictly monomorphic nature and a closed pan-genomic structure (Milani et al., 2013). We speculate that *B. animalis* subsp. *lactis* originated from the animal gut and subsequently colonized the human gut through ubiquitous environmental pollution, with transplant adaption between three ecological niches (human gut, environment, and animal gut) eroding genetic specialization and phylogenetic signal.

Nevertheless, according to our and other studies, the population structure of human-derived strains, including nomadic lifestyle *Lactobacillus* species, displays, to some extent, clustering by isolation origin, with isolates from children of the same ethnic groups being clustered throughout the dendrogram (Yuan et al., 2022). Autochthonous organisms could establish stable populations of typical sizes over long periods and exert specific ecological functions in a host habitat (Duar et al., 2017). In terms of adaptation to the human host gut, the autochthonous strain from a specific population as a probiotic should possess an innate advantage over strains from other sources. Indeed, some gut probiotic strains were shown to possess adaptive features to niches associated with humans that contribute to their persistence, pointing out the importance of adaptation to human hosts for the development of probiotics for humans (Barratt et al., 2022). Yet, the symbiosis and host specificity of specific bacterial symbionts in the human gut microbiome remain poorly understood.

In summary, *Bifidobacteria* are a paragon of symbiotic bacteria biota in the human gut. Evidence from symbiotic strain level analysis supports the vertical transmission of *Bifidobacterium* phylotypes from mother to offspring. Some well-known *Bifidobacterium* species or subspecies showed triadic synchronism in mother–milk–infant triads. Conspecific strains of *Bifidobacteria* of the same mother–child pair belong to the same monophyletic group and are genetically and physiologically very similar. *B. longum* subsp. *longum* and *B. breve* strains have considerable genetic heterogeneity across three ethnic groups and were clustered into ethnic-specific lineages, while all strains of *B. animalis* subsp. *lactis* present in three mother–infant cohorts displayed high homogeneity. *Bifidobacterium* species with distinct lifestyles differ strikingly in phylogenetic diversity, influencing the symbiotic association of these strains with the human host. Further whole-genome sequencing is required to provide in-depth insight into the genetic diversity, phylogeny, and population structure of *Bifidobacterium*.

## Data availability statement

The data presented in the study are deposited in the GenBank database under accession numbers PP768172 to PP768217 and PP780522 to PP780548.

## Ethics statement

Human volunteers involved in this study were reviewed and approved by the Ethics Committee of the First Affiliated Hospital, Shihezi University School of Medicine (2017-117-01). The informed consent was provided to participants before this study.

## Author contributions

SA: Data curation, Formal analysis, Investigation, Methodology, Writing – original draft, Writing – review & editing. ZL: Data curation, Formal analysis, Investigation, Methodology, Writing – original draft, Writing – review & editing. XZ: Data curation, Methodology, Writing – review & editing. BL: Resources, Writing – review & editing. HZ: Resources, Writing – review & editing. JH: Data curation, Software, Writing – review & editing. FT: Funding acquisition, Writing – review & editing. HS: Conceptualization, Methodology, Writing – review & editing. YN: Conceptualization, Funding acquisition, Writing – review & editing.
