# Supplementary material for: Genetic specialization of key bifidobacterial phylotypes in multiple mother–infant dyad cohorts from geographically isolated populations
Source: Front Microbiol. 2024 Jul 3;15:1399743. doi: 10.3389/fmicb.2024.1399743 (PMC11251887; doi:10.3389/fmicb.2024.1399743)
Supplement: Supplementary file 1 [file Data_Sheet_1.docx]

**Data sheet**

| Housekeeping-gene | Primer | Sequence(5’-3’) | Amplification product  position on the gene | Amplicon  size (bp) | Annealing temperature(℃) |
| --- | --- | --- | --- | --- | --- |
| *gyrB* | gyrBB3  gyrBB4 | AGCTGCACGCBGGCGGCAAGTTCG  GTTGCCGAGCTTGGTCTTGGTCTG | 383-1194 | 811 | 58 |
| *rplB* | rplBBB3  rplBBB4 | GGACAAGGACGGCRTSCCSGCCAA  ACGACCRCCGTGCGGGTGRTCGAC | 210-708 | 498 | 58 |
| *fusA* | fusAB3  fusAB4 | ATCGGCATCATGGCYCACATYGAT  CCAGCATCGGCTGMACRCCCTT | 40-824 | 784 | 58 |
| *ileS* | ileSB3  ileSB4 | ATCCCGCGYTACCAGACSATG  CGGTGTCGACGTAGTCGGCG | 253-1042 | 789 | 53 |
| *clpC* | ClpC-uni  ClpC-rev | GAGTACCGCAAGTACATCGAG  CATCCTCATCGTCGAACAGGAAC | 991-1735 | 744 | 58 |
| *purF* | PurF-uni  PurF-rev | CATTCGAACTCCGACACCGA  GTGGGGTAGTCGCCGTTG | 400-1376 | 976 | 58 |
| *rpoB* | rpoBB3  rpoBB4 | GGCGAGCTGATCCAGAACCA  GCATCCTCGTAGTTGTASCC | 1135-2192 | 1057 | 53 |

**Table S1:** Primers for amplification of housekeeping genes

**Table S2:** Descriptive analysis of allele sequence information and diversity of MLST

π, the mean pairwise nucleotide differences per site.

dN/dS, ratio of nonsynonymous to synonymous mutations.

| Gene | Number of alleles | G+C % | π^a^ | dN/dS^b^ | Tajima‘s D | Phi-test |
| --- | --- | --- | --- | --- | --- | --- |
| *gyrB* | 12 | 60.6 | 0.15028 | 0.5174 | -0.82485 | <0.001 |
| *rplB* | 8 | 63.9 | 0.09725 | 1.6836 | -0.93550 | <0.001 |
| *fusA* | 12 | 58.9 | 0.10718 | 2.8616 | -0.17492 | <0.001 |
| *ileS* | 15 | 61.7 | 0.08196 | 0.1482 | -1.17651 | <0.001 |
| *clpC* | 7 | 60.9 | 0.09687 | 1.5333 | 3.03016 | <0.001 |
| *purF* | 9 | 62.2 | 0.09067 | 0.5429 | 2.63015 | <0.001 |
| *rpoB* | 10 | 62.0 | 0.06716 | 0.6245 | -1.88299 | <0.001 |
