# Supplementary material for: Genetic specialization of key bifidobacterial phylotypes in multiple mother–infant dyad cohorts from geographically isolated populations
Source: Front Microbiol. 2024 Jul 3;15:1399743. doi: 10.3389/fmicb.2024.1399743 (PMC11251887; doi:10.3389/fmicb.2024.1399743)
Supplement: Supplementary file 2 [file Data_Sheet_2.docx]

**Supplementary Material**

**Supplementary Figures**

**Figure S1:** Phylogenetic dendogram and rep-PCR band pattern for the 100 representative *Bifidobacterium* isolates. Percent similarity was calculated using the Pearson correlation coefficient. The clustering was performed using the UPGMA. （**A**）, *B.longum* subsp. *longum* ; （**B**）, *B.breve*; （**C**）, *B.animalis* subsp. *lactis.*

**Figure S2:** Neighbour Joining (NJ) trees of the individual genes (*clpC, fusA, gyrB, ileS, purF,rplB rpoB*) were constructed in MEGA.

**Figure. S3:** Split-decomposition analysis of individual MLST loci

**Supplementary tables**

**Table S1:** Primers for amplification of housekeeping genes

**Table S2:** Descriptive analysis of allele sequence information and diversity of MLST

π, the mean pairwise nucleotide differences per site.

dN/dS, ratio of nonsynonymous to synonymous mutations.
