# Supplementary figures and images for: Genetic specialization of key bifidobacterial phylotypes in multiple mother–infant dyad cohorts from geographically isolated populations
Source: Front Microbiol. 2024 Jul 3;15:1399743. doi: 10.3389/fmicb.2024.1399743 (PMC11251887; doi:10.3389/fmicb.2024.1399743)

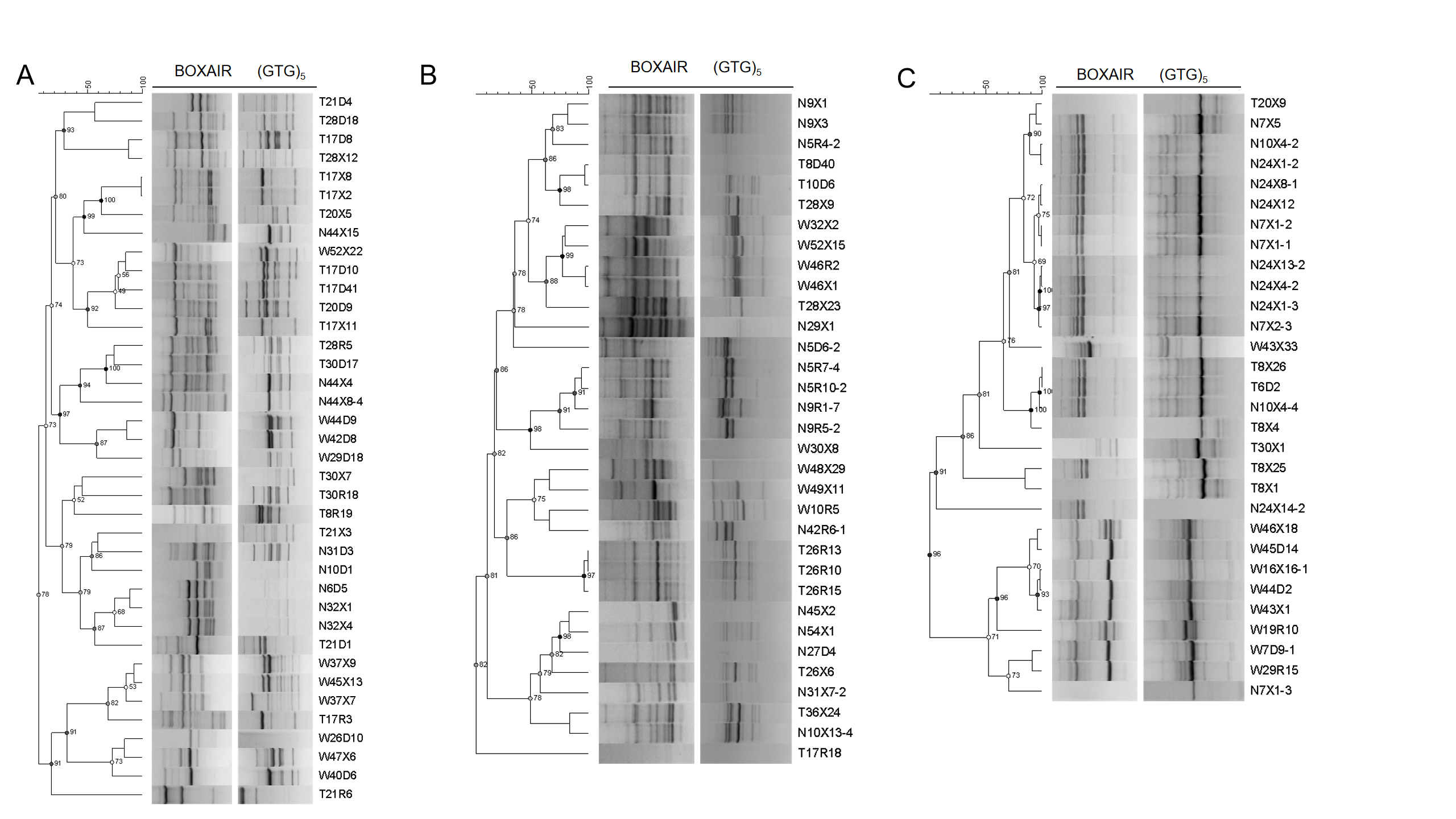

Supplement: Supplementary file 3 [file Image_1.TIF]

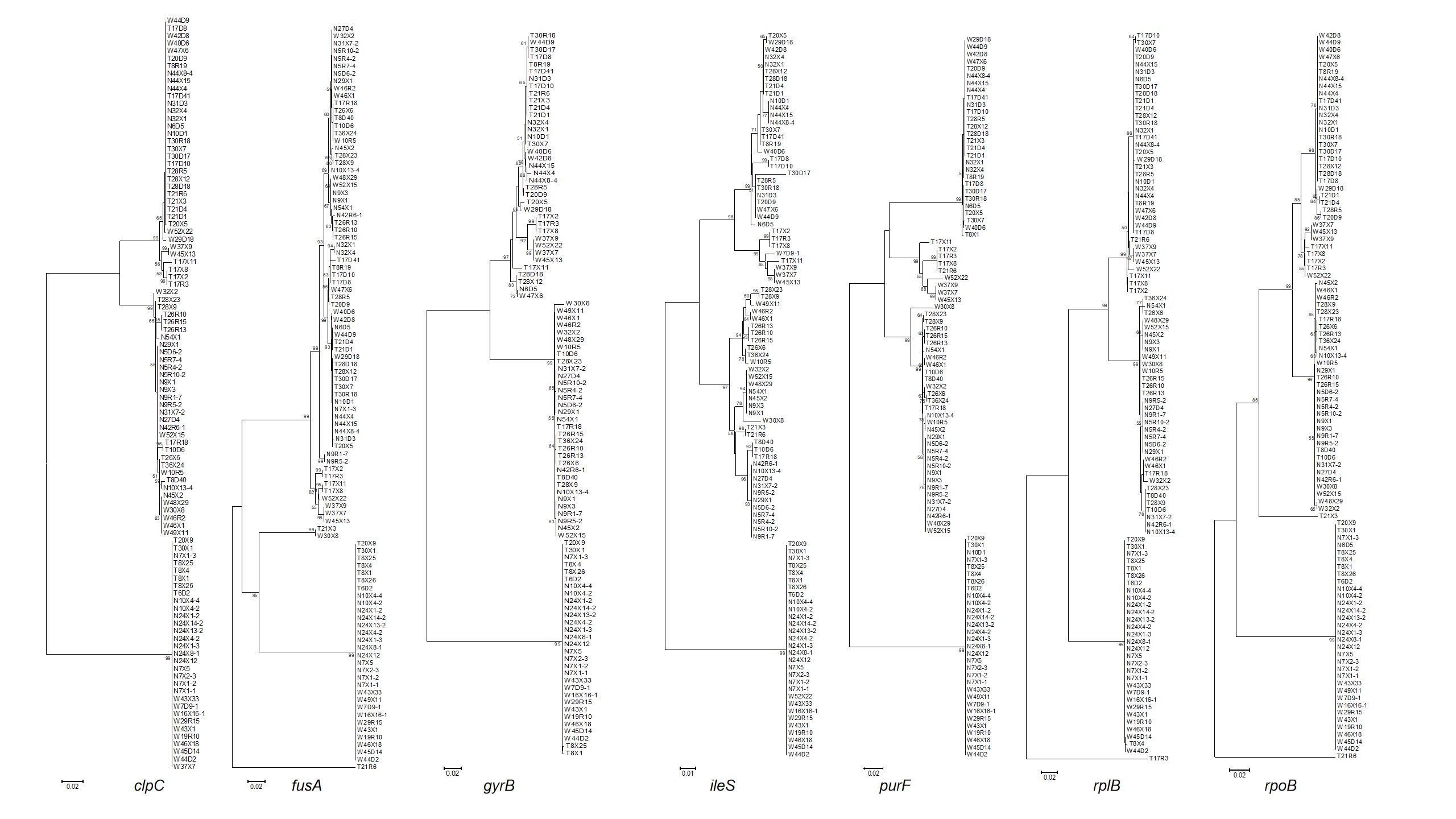

Supplement: Supplementary file 4 [file Image_2.TIF]

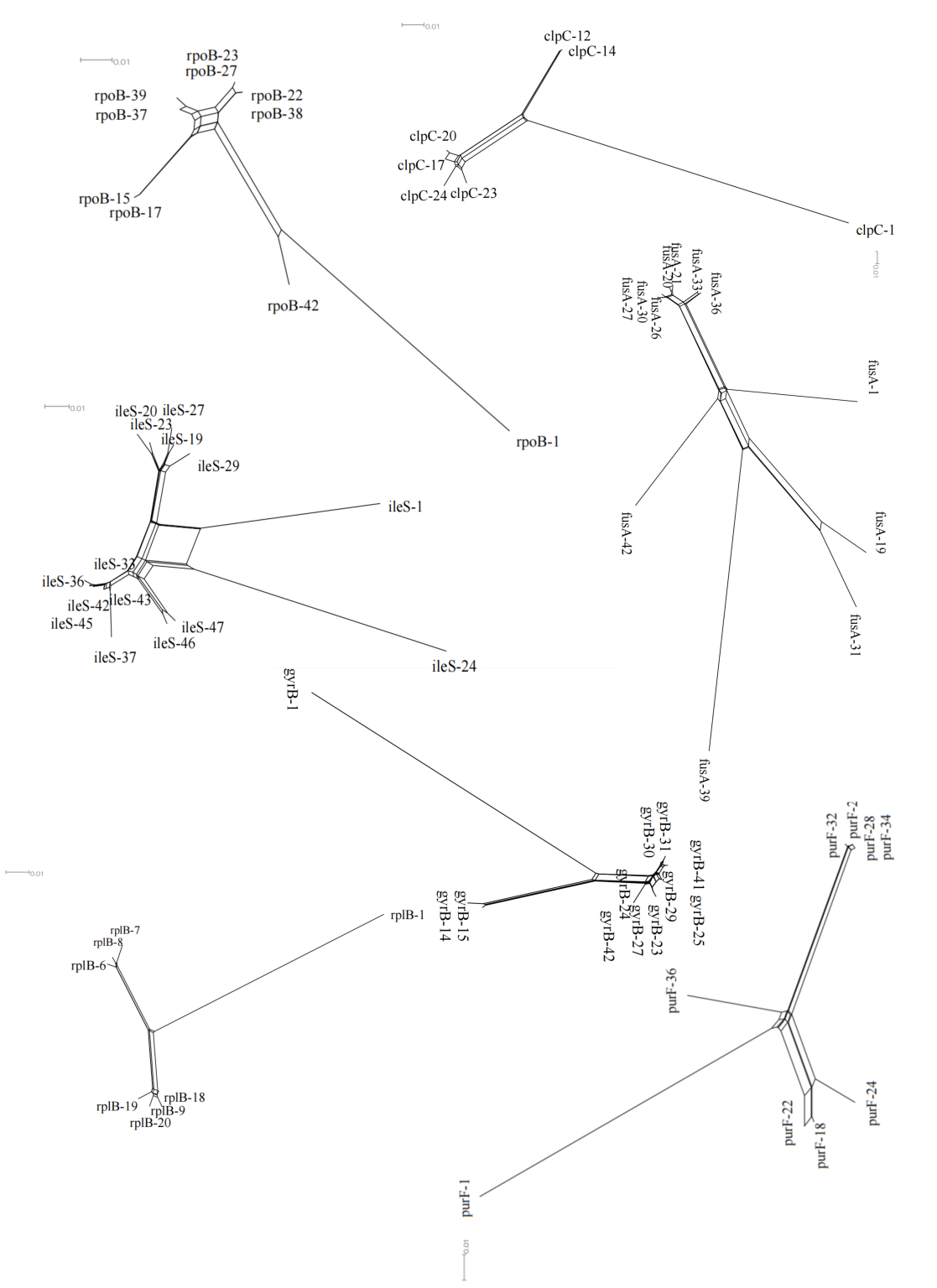

Supplement: Supplementary file 5 [file Image_3.TIF]
